# Hypothermia related to continuous renal replacement therapy: incidence and associated factors

**DOI:** 10.5935/0103-507X.20210012

**Published:** 2021

**Authors:** Cássia Maria Frediani Morsch, Jaqueline Sangiogo Haas, Rose Plotnick, Taciana de Castilhos Cavalcanti, Patrícia Cristina Cardoso, Tatiana Pilger, Juliana Teixeira da Silveira, Fernando Saldanha Thomé

**Affiliations:** 1 Intensive Care Center, Hospital de Clínicas de Porto Alegre, Universidade Federal do Rio Grande do Sul - Porto Alegre (RS), Brazil.

**Keywords:** Renal dialysis, Hemodiafiltration, Hypothermia, Incidence, Risk factors, Intensive care units, Diálise renal, Hemodiafiltração, Hipotermia, Incidência, Fatores de risco, Unidades de terapia intensiva

## Abstract

**Objective:**

To evaluate the incidence of hypothermia in patients undergoing continuous renal replacement therapy in the intensive care unit. As secondary objectives, we determined associated factors and compared the occurrence of hypothermia between two modalities of continuous renal replacement therapy.

**Methods:**

A prospective cohort study was conducted with adult patients who were admitted to a clinical-surgical intensive care unit and underwent continuous renal replacement therapy in a high-complexity public university hospital in southern Brazil from April 2017 to July 2018. Hypothermia was defined as a body temperature ≤ 35ºC. The patients included in the study were followed for the first 48 hours of continuous renal replacement therapy. The researchers collected data from medical records and continuous renal replacement therapy records.

**Results:**

A total of 186 patients were equally distributed between two types of continuous renal replacement therapy: hemodialysis and hemodiafiltration. The incidence of hypothermia was 52.7% and was higher in patients admitted for shock (relative risk of 2.11; 95%CI 1.21 - 3.69; p = 0.009) and in those who underwent hemodiafiltration with heating in the return line (relative risk of 1.50; 95%CI 1.13 - 1.99; p = 0.005).

**Conclusion:**

Hypothermia in critically ill patients with continuous renal replacement therapy is frequent, and the intensive care team should be attentive, especially when there are associated risk factors.

## Abstract (pt)

**Objetivo:**

Avaliar a incidência de hipotermia em pacientes em terapia renal substitutiva contínua na unidade de terapia intensiva. Como objetivos secundários, determinar fatores associados e comparar a ocorrência de hipotermia entre duas modalidades de terapia renal substitutiva contínua.

**Métodos:**

Estudo de coorte, prospectivo, realizado com pacientes adultos internados em uma unidade de terapia intensiva clínico-cirúrgica, que realizaram terapia renal substitutiva contínua em um hospital universitário público de alta complexidade do Sul do Brasil, de abril de 2017 a julho de 2018. A hipotermia foi definida como queda da temperatura corporal ≤ 35ºC. Os pacientes incluídos no estudo foram acompanhados nas 48 horas iniciais de terapia renal substitutiva contínua. Os dados foram coletados pelos pesquisadores por meio da consulta aos prontuários e às fichas de registro das terapias renais substitutivas contínuas.

**Resultados:**

Foram avaliados 186 pacientes distribuídos igualmente entre dois tipos de terapia renal substitutiva contínua: hemodiálise e hemodiafiltração. A incidência de hipotermia foi de 52,7%, sendo maior nos pacientes que internaram por choque (risco relativo de 2,11; IC95% 1,21 - 3,69; p = 0,009) e nos que fizeram hemodiafiltração com aquecimento por mangueira na linha de retorno (risco relativo de 1,50; IC95% 1,13 - 1,99; p = 0,005).

**Conclusão:**

A hipotermia em pacientes críticos com terapia renal substitutiva contínua é frequente, e a equipe intensivista deve estar atenta, em especial quando há fatores de risco associados.

## INTRODUCTION

The incidence of acute kidney injury (AKI) in intensive care unit (ICU) patients has increased worldwide and varies from 20% to 50% depending on the definition used and the study population.^(1)^

The use of renal replacement therapy (RRT), which may be intermittent or continuous, is increasing in ICUs.^(2,3)^ Continuous RRT (CRRT) is mainly indicated for hemodynamic instability and the risk of increased intracranial pressure.^(4,5)^

The choice of CRRT method includes continuous venovenous hemodialysis (CVVHD), continuous venovenous hemofiltration, continuous venovenous ultrafiltration, and continuous venovenous hemodiafiltration (CVVHDF) and depends on the clinical status of the patient, the technical skills, and the availability of supplies at each institution.^(6-8)^

The possibility of adverse events in RRT, including hypotension, arrhythmias, and hypothermia, may reach 97%. Hypothermia (body temperature < 35ºC) in CRRT occurs from heat loss due to extracorporeal blood circulation. This condition is insufficiently diagnosed and may occur in 44% of cases.^(4)^

The care team should be aware of this complication because thermal instability can potentially mask ongoing sepsis, mimic bacteremia, induce fever and chills, and predispose patients to arrhythmias and hemodynamic instability.^(9,10)^

Hypothermia in CRRT is recognized worldwide as a clinical complication; however, published studies on this topic are scarce. Thus, we conducted a prospective observational study with the primary objective of evaluating the incidence of hypothermia in our patients with CRRT. As secondary objectives, we sought to determine related factors and compare the occurrence of hypothermia between our two treatment modalities, CVVHD and CVVHDF.

## METHODS

A prospective cohort study was conducted with adult patients admitted to a 39-bed clinical-surgical ICU of a high-complexity public university hospital in southern Brazil from April 2017 to July 2018. The inclusion criteria were as follows: patients older than 18 years who underwent continuous extracorporeal dialysis methods and had AKI or chronic kidney disease and were not previously hypothermic.

Patients with other types of extracorporeal therapy, such as extracorporeal membrane oxygenation and plasmapheresis, those with contraindications to the insertion of an esophageal thermometer, and those for whom therapy was interrupted for more than 6 hours during follow-up or ended before 24 hours of treatment were excluded.

The criteria for sepsis and septic shock used in the study were based on the definition of the Third International Consensus Definitions for Sepsis and Septic Shock (Sepsis-3).^(11)^ The dose of norepinephrine used was classified as low (dose < 0.2mcg/kg/minute), moderate (dose from 0.2 to 0.5mcg/kg/minute) and high (dose > 0.5mcg/kg/minute). To define nutritional status, the following body mass index (BMI) classifications were used: underweight for BMI < 18.5kg/m^2^, normal weight for BMI between 18.5kg/m^2^ and 24.9kg/m^2^, overweight for BMI between 25kg/m^2^ and 29.9kg/m^2^, and obese for BMI > 30.0 kg/m^2^.

The patients included in the study were followed for the first 48 hours of CRRT.

Continuous venovenous hemodialysis was performed with a Diapact^®^ device and extracorporeal circuit with a priming volume (filling volume) of 300mL. The heating system was operated by passing the dialysate solution through a plate that heated it to a temperature of 39°C as a routine dialysis prescription.

Continuous venovenous hemodiafiltration with postfilter replacement solution was performed with a Prismaflex device and extracorporeal circuit with a priming volume of 150mL. The heating system used was Prismaflo^®^, in which a thermal hose was adapted to the venous line (blood return to the patient) to heat the blood return to a fixed temperature of 39ºC as a routine dialysis prescription protocol.

The commercial dialysate and replacement solutions used were in 5,000mL plastic bags containing Hemolenta (Eurofarma) or electrolyte solution for dialysis from the Life® laboratory. All patients had dialysis access through a 12F double- or triple-lumen catheter, which was typically inserted in the right internal jugular vein (length 15cm - 16cm; 40 ± 8%) or femoral vein (length 24cm - 30cm; 34 ± 9.4%). The blood flow rate prescribed for adult patients was 150mL/minute, according to the institution’s routine protocol.

All patients underwent regional anticoagulation with 4% trisodium citrate, which was infused directly into the (arterial) access line of the extracorporeal circuit. Replacement was performed with calcium gluconate solution, which was infused into a central access line different from the dialysis access line.

The choice of CRRT method was chosen by the nephro-intensive care physician, according to the patient’s needs for clearance and ultrafiltration. The prescribed dialysis dose usually varies between 25mL/kg/hour and 35mL/kg/hour.

The ICU environment was air-conditioned, with a constant temperature between 22ºC and 23ºC.

According to the routine protocol of the unit, the axillary body temperature was measured with a standardized digital thermometer at the institution and recorded every 2 hours. Specific cases involved continuous measurement of blood temperature by a Swan-Ganz catheter. For patients in whom the axillary temperature was measured, an esophageal thermometer was installed for more accurate monitoring (according to the institutional standard operating procedure) in the event of a temperature ≤ 35ºC. Hypothermia was defined as a decrease in body temperature (temperature ≤ 35ºC) and classified as mild (temperature between 35ºC and 32ºC), moderate (temperature between 32ºC and 28ºC) or severe (temperature below 28ºC).^(12)^ All patients with hypothermia were heated with a thermal blanket, according to the institution’s routine protocol.

To calculate the sample size, WinPepi software, version 11.43, was used. Considering a power of 80%, significance level of 5% and difference between incidences of hypothermia of 20% in the different dialysis methods, as reported by Yagi et al.,^(13)^ a total sample size of 186 patients was obtained.

The researchers collected data from medical records and dialysis records and transferred them to an Excel spreadsheet, which was later exported and analyzed using the Statistical Package for the Social Sciences (SPSS), version 18.

Continuous variables were expressed as the mean and standard deviation, and categorical variables were expressed as absolute and relative frequencies. The normality of the continuous variables was verified by the Shapiro-Wilk test. Continuous variables were compared by unpaired Student’s t-tests, and categorical variables were compared by Pearson’s chi-square tests. The time until the first hypothermia episode for the groups was evaluated by Kaplan-Meier curves and compared by the log-rank test. Because this study was longitudinal with a dichotomous outcome, Poisson regression with adjustment for robust variances was used to evaluate independent associations with hypothermia. Sex, Simplified Acute Physiology Score (SAPS III) and age were included by clinical significance. The method used was Enter. The accuracy of the model was evaluated using the receiver operating characteristic (ROC) curve. The results were considered statistically significant for p < 0.05 with a 95% confidence interval (CI).

This study was approved by the Research Ethics Committee of the institution under protocol 20170009 and met the precepts of resolution 466/12 of the National Health Council. The patients or their representatives signed the Terms of the Free and Informed Consent form authorizing the use of data from the patients’ records.

## RESULTS

During the study period, 186 patients were equally distributed between the two types of CRRT. The overall mean age was 57.8 ± 16.5 years, and patients were predominantly male (60.2%) and white (82.6%). Most patients were clinical (66.1%), and the most frequent reason for hospitalization was shock (54.3%), followed by acute respiratory failure (ARF; 23.1%). Sepsis or septic shock was present in 70% of cases. A proportion of 78% of patients underwent invasive mechanical ventilation (IMV), and 79% received vasoactive drugs at the onset of CRRT. The overall mean SAPS III was 72.9 ± 18.6 (Table 1). The mean patients follow-up time was 45.8 ± 5.3 hours from the beginning of dialysis therapy, with a minimum of 24 hours and a maximum of 48 hours.

**Table 1:** Sociodemographic and clinical characteristics of patients who underwent continuous renal replacement therapy (continuous venovenous hemodialysis or continuous venovenous hemodiafiltration)

	Total number of patients (n = 186)	CVVHD (96; 51.6%)	CVVHDF (90; 48.4%)	p value
Age	57.8 ± 16.5	58.9 ± 16.6	56.5 ± 16.5	0.306†
Male sex	112 (60.2)	57 (59.4)	55 (61.1)	0.927‡
SAPS III	72.9 ± 18.6	70.9 ± 19.8	74.9 ± 17.1	0.149†
BMI				
Underweight	3 (1.6)	3 (3.1)	0	
Normal weight	59 (31.9)	35 (36.6)	24 (27)	
Overweight	58 (31.4)	36 (37.6)	22 (24.7)	
Obese	65 (35.1)	22 (22.9)	43 (35.1)	< 0.002‡
Reason for ICU admission				
ARF	43 (23.1)	23 (24)	20 (22.2)	
Shock	101 (54.3)	50 (52.1)	51 (56.7)	
Other	43 (22.6)	23 (22.8)	20 (20.0)	0.649‡
Comorbidity				
Cirrhosis	17 (9.1)	4.0 (4.2)	13 (14.4)	0.030‡
Hypothyroidism	7 (3.8)	5 (5.2)	2 (2.2)	0.494‡
Adrenal insufficiency	2 (1.1)	1 (1)	1 (1.1)	0.963‡
Type of patient				
Clinical	123 (66.1)	62 (65.3)	61 (67.8)	0.837‡
Surgical	62 (33.3)	33 (34.7)	29 (32.2)	
Sepsis				
No	56 (30.1)	30 (31.3)	26 (28.9)	
Sepsis	16 (8.6)	7 (7.3)	9 (10)	
Septic shock	114 (61.3)	59 (61.5)	55 (61.1)	0.785‡
Ventilation				
Spontaneous	33 (17.7)	18 (18.8)	15 (16.7)	
Invasive	145 (78)	72 (75)	73 (81.1)	0.498‡
Noninvasive	8 (4.3)	6 (6.2)	2 (2.2)	
Vasopressor				
No	39 (21)	24 (25)	15 (16.7)	
Low	58 (31.2)	26 (27)	32 (35.6)	0.453‡
Moderate	47 (25.3)	24 (25)	23 (25.6)	
High	42 (22.6)	22 (22.9)	20 (22.2)	
Hypothermia	98 (52.7)	40 (40.8)	58 (59.2)	0.003‡
Mortality	92 (49.5)	51 (53.1)	53 (47.8)	0.560‡

CVVHD - continuous venovenous hemodialysis; CVVHDF- continuous venovenous hemodiafiltration; SAPS III - Simplified Acute Physiology Score III; BMI - body mass index; ICU - intensive care unit; ARF - acute respiratory failure. †values expressed as the mean ± standard deviation compared by Student's t-test; ‡values expressed as n (%) compared by Pearson's chi-square test.

The patients underwent two types of therapy: 96 (51.6%) underwent CVVHD with heating of the dialysate, and 90 (48.4%) underwent CVVHDF with heating of the return line. The two groups were similar regarding their sociodemographic and clinical characteristics and mortality rate. However, in the group that underwent CVVHDF, there were more patients with high BMI (35.1% *versus* 22.9%; p < 0.002) and cirrhosis (14.4% *versus* 4.2%; p = 0.030). This difference was expected because the institutional protocol indicated CVVHDF for obese and cirrhotic patients.

More than half of the patients on continuous therapy exhibited hypothermia during follow-up (52.7%), as shown in table 1. Hypothermia was mild (between 35°C and 32°C) in the majority of patients. Only one patient on CVVHDF experienced moderate hypothermia (31.9°C).

In addition to the factors related to dialysis therapy, we sought other factors that could be associated with the development of hypothermia. Patients who were hospitalized due to shock (66.3% *versus* 40.9%; p < 0.001), had septic shock (71.4% *versus* 50%; p = 0.011), or received vasopressor drugs at any dose (p = 0.029) experienced hypothermia more frequently. Patients who underwent IMV (85.7% *versus* 69.3%, p = 0.027) and those who had the worst outcome (death) had more hypothermia (58.2% *versus* 39.8%, p = 0.018) (Table 2).

**Table 2:** Characteristics of hypothermic and nonhypothermic patients

	Nonhypothermic (88; 47.3%)	Hypothermic (98; 52.7%)	p value
Age	58.5 ± 15.3	57.1 ± 17.6	0.566*
SAPS III	72.1 ± 17.5	73.6.4 ± 19.7	0.590*
Male sex	58 (65.9)	54 (55.1)	0.176†
BMI			
Underweight	2 (2.3)	1 (1.0)	
Normal weight	28 (31.8)	31 (32.0)	
Overweight	28 (31.8)	30 (30.9)	
Obese	30 (34.1)	35 (36.1)	0.918†
Reason for ICU admission			
ARF	21 (23.9)	22 (22.4)	
Shock	36 (40.9)	65 (66.3)	<0.001†
Other	31 (35.2)	11 (11.2)	
Comorbidity			
Cirrhosis	10 (11.4)	7 (7.1)	0.458†
Hypothyroidism	5 (5.7)	2 (2)	0.359†
Adrenal insufficiency	1 (1.1)	1 (1)	0.939†
Type of patient			
Clinical	56 (63.6)	67 (69.1)	0.434†
Surgical	32 (36.4)	30 (30.9)	
Sepsis			
No	34 (38.6)	22 (22.4)	
Sepsis	10 (11.4)	6 (6.1)	
Septic shock	44 (50)	70 (71.4)	0.011†‡
Ventilation			
Spontaneous	20 (22.7)	13 (13.3)	
Invasive	61 (69.3)	84 (85.7)	0.027†‡
Noninvasive	7 (7.9)	1 (1)	
Vasopressor			
No	26 (29.5)	13 (13.3)	
Low	28 (31.8)	30 (30.6)	0.029†‡
Moderate	17 (19.3)	30 (30.6)	
High	17 (19.3)	25 (25.5)	
Mortality	35 (39.8)	57 (58.2)	0.018†‡

SAPS III - Simplified Acute Physiology Score III; BMI - body mass index; ICU - intensive care unit; ARF - acute respiratory failure.

* Values expressed as the mean ± standard deviation compared by Student's t-test †values expressed as n (%) compared by Pearson's chi-square test; ‡p <0.05.

There was no statistically significant difference in the mean pretreatment temperature between the two groups. The highest incidence of hypothermia occurred in patients on CVVHDF with heating of the return line (59.2% *versus* 40.8%; p = 0.003), which also showed the greatest variations and the lowest temperatures in the study (Table 3).

**Table 3:** Comparison between the hypothermia and temperature groups in continuous renal replacement therapy

	CVVHD (n = 96)	CVVHDF (n = 90)	p value
Hypothermia during dialysis	40 (41.7)	58 (64.4)	0.003* ‡
Temperature pre-CRRT	36.4 ± 0.66	36.6 ± 0.92	0.222†
Lower temperature	35.1 ± 0.79	34.6 ± 0.92	< 0.001*†
Temperature variation (ºC)	1.35 ± 0.89	1.98 ± 1.24	< 0.001*†

CVVHD - continuous venovenous hemodialysis; CVVHDF - continuous venovenous hemodiafiltration; CRRT - continuous renal replacement therapy.

* p < 0.05; †values expressed as the mean ± standard deviation compared by Student's t-test; ‡values expressed as n (%) compared by Pearson's chi-square test.

The median time between the onset of CRRT and hypothermia was 8 hours (4 - 8 hours) in the CVVHD group and 6 hours (4 - 6 hours) in the CVVHDF group. There was no significant difference between the two groups (p = 0.449).

**Figure 1:**
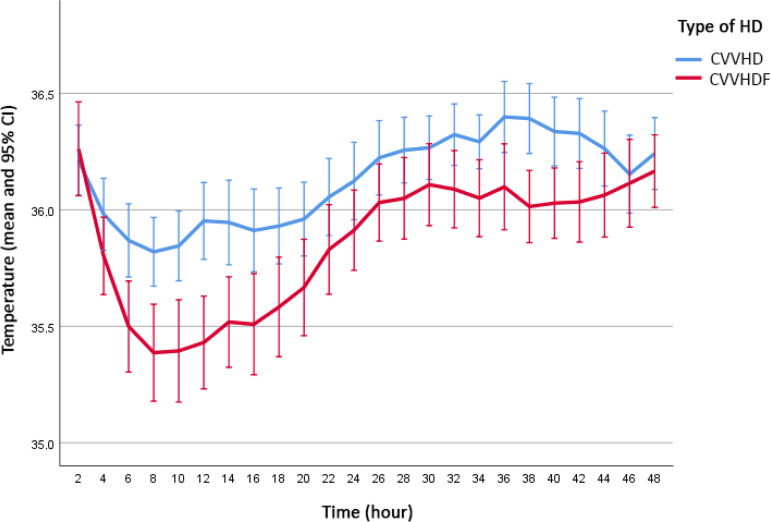
Mean temperatures during follow-up.

Figure 1 shows the evolution of the mean temperatures in both groups during follow-up. Lower temperatures were observed in the group of patients on CVVHDF with heating of the return line. The gradual increase in temperature of patients in both groups after hypothermia was related to the implementation of heating with thermal blankets.

95% CI - 95% confidence interval; HD - hemodialysis; CVVHD - continuous venovenous hemodialysis; CVVHDF - continuous venovenous hemodiafiltration.

We sought to evaluate whether the replacement solution flow rate, a component of CVVHDF therapies and not used in CVVHD, was a factor related to the higher incidence of hypothermia. The replacement flow rate varied from 100 to 1,500mL/hour (mean = 673 ± 326mL/hour), but contrary to what we expected, this factor did not impact the development of hypothermia (r = -0.110; p = 0.273).

**Figure 2:**
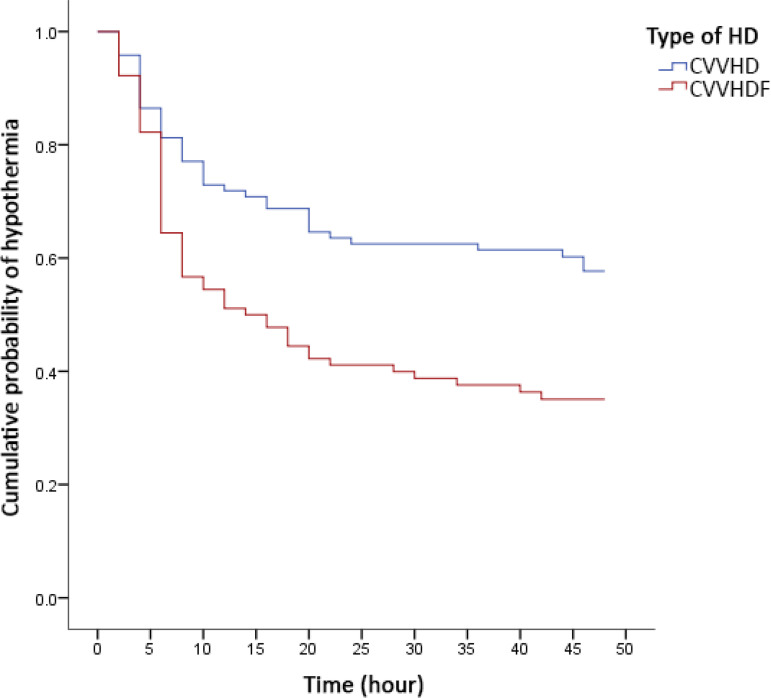
Probability of hypothermia.

Figure 2 shows the cumulative probability of hypothermia according to the dialysis/heating method used. The curves illustrate the time until the first hypothermia episode. Patients on CVVHDF with heating of the return line experienced hypothermia earlier than those on CVVHD with heating of the dialysate (p = 0.001).

HD - hemodialysis; CVVHD - continuous venovenous hemodialysis; CVVHDF - continuous venovenous hemodiafiltration; Kaplan-Meier curves compared by the log-rank test; p = 0.001.

Multiple regression analysis (Poisson regression) was performed to determine the importance of factors associated with hypothermia. Regarding the type of CRRT, CVVHDF had a relative risk (RR) of 1.50 and 95%CI of 1.13 - 1.99, with p = 0.005. Hospitalization due to shock had an RR of 2.11 and 95%CI of 1.21 - 3.69, with p = 0.009. ARF, IMV and sepsis lost statistical power (Table 4). The accuracy of the model, which was evaluated using the ROC curve, showed an area under the curve of 0.753.

**Table 4:** Multiple Poisson regression of the continuous renal replacement therapy adjusted for the other associated factors in relation to hypothermia

	RR	95% CI	p value
Reason for ICU admission			
Shock	2.11	(1.21 - 3.69)	0.009*
ARF	1.75	(0.96 - 3.18)	
Other	1		
Use of vasopressors	1.12	(0.68 - 1.85)	0.650
Sepsis	1.04	(0.74 - 1.47)	0.825
Male sex	0.79	(0.61 - 1.02)	0.068
SAPS III	0.99	(0.99 - 1.01)	0.862
Age	0.99	(0.99 - 1.01)	0.712
IMV	1.38	(0.89 - 2.14)	0.155
CVVHDF	1.50	(1.13 - 1.99)	0.005*

RR - relative risk; 95% CI - 95% confidence interval; ICU - intensive care unit; ARF - acute respiratory failure; SAPS III - Simplified Acute Physiology Score III; IMV - invasive mechanical ventilation; CVVHD - continuous venovenous hemodialysis; CVVHDF - continuous venovenous hemodiafiltration.

* p < 0.05; multiple Poisson regression with adjustment.

## DISCUSSION

Our study, which considered hypothermia at temperatures of 35ºC or lower, showed an overall incidence of hypothermia of 57.2%. We sought to compare the incidence between two different dialysis methods (diffusive and diffusive/convective) with different equipment and different heating systems but with exclusively venovenous access. In our study, there was a higher incidence of hypothermia in patients who underwent CVVHDF (59.2%) *versus* CVVHD (40.8%), with p = 0.003. In the Poisson regression, one of the CRRT methods used (CVVHDF; p = 0.005) and admission to the ICU due to shock (p = 0.009) were factors associated with hypothermia.

In our literature review, one of the first studies to address hypothermia during CRRT was conducted by Yagi et al.^(13)^ in 1998; in this study, the incidence of hypothermia (temperature < 35.5 °C) was 38% and included 51% of patients in each phase of the study. Akhoundi et al.^(4)^ evaluated adverse events in CRRT; the incidence of hypothermia (temperature < 35ºC) in their study was 44%. Moreover, Rickard et al.^(14)^ exclusively evaluated patients on CVVHDF and found no significant difference in the incidence of hypothermia (temperature < 36ºC) with heating of the solution (34.6%) and without heating of the solution (40%). According to Ricci and Romagnoli,^(15)^ up to 90% of patients undergoing CRRT may experience hypothermia.

Unlike the results found in this study, studies in the literature suggests other factors associated with hypothermia in CRRT.^(4,13,14)^ Yagi et al.^(13)^ showed that lower body weight (79.8 ± 19 *versus* 88.6 ± 19.9; p = 0.04) was associated with hypothermia and that a lower blood flow rate or higher dialysis solution flow rate resulted in a greater loss of energy and lower body temperature. We found no statistically significant difference in the BMI of hypothermic patients compared to those who retained a normal body temperature. However, in the group that underwent CVVHDF, there were more patients with high BMI (43% *versus* 22%; p < 0.002), and this group had more hypothermia. It should be noted that the weight used to calculate the BMI was the patient’s weight at the time of ICU admission, so high values may be affected by a hypervolemic state.

The higher occurrence of hypothermia in patients from the study by Yagi et al.^(13)^ was associated with venovenous modalities due to the greater length of the extracorporeal circuit and its greater volume of blood filling than the circuit of the arteriovenous modalities. The largest filling volume was not associated with hypothermia in our study because the extracorporeal circuit operated with 300mL in CVVHD, while the CVVHDF circuit operated with 150mL, and the latter was associated with a higher incidence of hypothermia. In the prospective phase of the study by Yagi et al.,^(13)^ CVVHD sessions were exclusively evaluated with a blood flow rate from 100mL/minute to 200 mL/minute and dialysis solution flow rate from 500mL/hour to 1,500mL/hour.

In our study, the blood flow rate was constant (150mL/minute) for all therapies, but there was great variability in the dialysis solution and replacement solution flow rates. Patients who were exclusively dialyzed using the diffusive method on equipment with heating of the dialysate solution had a lower incidence of hypothermia. However, we found no relationship between replacement flow rate and lower temperatures by analyzing only our patients on CVVHDF.

In the study by Rickard et al.,^(14)^ female sex was a powerful predictor of hypothermia, with an RR of 0.185, 95%CI of 0.060 - 0.573 and p = 0.003. In the study by Yagi et al.^(13)^, there was no difference in the severity score between the groups of hypothermic and nonhypothermic patients according to the Acute Physiology and Chronic Health Evaluation (APACHE II) score. In our study, female sex and severity scores were not associated with hypothermia.

The general characteristics of the patients in our study were similar to those found for patients in the literature: male sex was predominant; other characteristics included hospitalization for clinical reasons, sepsis, vasopressor drugs, and mechanical ventilation.^(4,6)^ The mean age of our patients was 58.3 ± 16.6 years, which was also similar to the age range found for patients in the literature.^(4,6)^

Patients with AKI undergoing RRT are among the most severe in ICUs. Different severity scores have been used to determine the mortality risk of patients in the ICU. In the study by Yagi et al.^(13)^, the APACHE II score was 19.2 ± 6.1, with a relatively low mortality risk (> 35%); in contrast, Rickard et al.^(14)^ found higher APACHE II scores (28.2 ± 8). Akhoundi et al.^(4)^ evaluated patients with a median APACHE III of 109 (91 - 130). Schefold et al.^(2)^ used SAPS II and found a mean of 63.8 ± 17.6. Except for the Yagi study, the mortality risk was similar (> 70%) for all studies, even though the severity was evaluated by different scores.^(16,17)^

In our study, patients were classified with SAPS III, with an overall mean of 72.9 ± 18.9 and risk of mortality between 50% and 60%, which is slightly lower than the scores found in the aforementioned studies.^(18)^ Contrary to what we expected, the severity of our patients measured by SAPS III did not allow us to differentiate patients at higher risk for hypothermia.

Despite technological and scientific advances in CRRT, patient mortality remains relatively constant over time at approximately 50%.^(19-21)^ In the study by Yagi et al.,^(13)^ mortality was high (74%) and was not associated with hypothermia. Our mortality rate in CRRT was 49.5% and was significantly higher in hypothermic patients (58.2% *versus* 39.8%; p = 0.018). Data from our study suggest that hypothermia is related to mortality because there was no difference in severity measured by SAPS III between the groups.

There are other factors related both to individuals and to external factors (sedation and immobility) that influence the thermoregulation of critically ill patients.^(14)^ One example is the patients who develop septic shock, a condition that has been associated with more extreme changes in body temperature, in which hypothermia has been attributed to a worse prognosis.^(22.23)^

Our study evaluated a topic rarely addressed in the literature and has positive aspects, such as the fact that it was prospective, included many patients, analyzed variables, and used an accurate methodology to measure temperature. The use of a thermal blanket was shown to be an efficient strategy to correct hypothermia.

### Study limitations

We found a difference in the incidence of hypothermia between two groups of patients with similar characteristics who underwent two different CRRT modalities. However, these therapies were performed using different equipment with different heating systems, which may have been a confounding factor.

Comparing our data to those of the few existing studies on the subject was a challenge. In previous studies, there were different severity indices of the patients, temperature values that defined hypothermia, CRRT methods and equipment, and variables analyzed.

Finally, the study was not powered to evaluate the risk factors with greater robustness, given the sample size and the low occurrence of some of these factors.

## CONCLUSION

Despite the heating system of the dialysis equipment, our study showed a high incidence of hypothermia, suggesting that one should be proactive in other ways to avoid hypothermia, especially if continuous venovenous hemodiafiltration is used.
